# Biosynthesis of Phenolic Compounds of *Medicago truncatula* After Inoculation with Selected PGPR Strains

**DOI:** 10.3390/ijms252312684

**Published:** 2024-11-26

**Authors:** Anna Kisiel, Tymoteusz Miller, Adrianna Łobodzińska, Kinga Rybak

**Affiliations:** 1 Institute of Marine and Environmental Sciences, University of Szczecin, Wąska 13, 71-415 Szczecin, Poland; tymoteusz.miller@usz.edu.pl; 2 Polish Society of Bioinformatics and Data Science BIODATA, Popiełuszki 4C, 71-214 Szczecin, Poland; 3 Faculty of Data Science and Information, INTI International University, Nilai 71800, Negeri Sembilan, Malaysia; 4 Institute of Biology, University of Szczecin, Wąska 13, 71-415 Szczecin, Poland; 5 Doctoral School of the University of Szczecin, 71-412 Szczecin, Poland

**Keywords:** phenylpropanoid biosynthesis pathway, phenylalanine ammonia-lyase (PAL), *Medicago truncatula*, total phenolic compound, plant growth-promoting rhizobacteria

## Abstract

The phenylpropanoid biosynthesis pathway is involved in the response of plants to stress factors, including microorganisms. This paper presents how free-living strains of rhizobacteria *Pseudomonas brassicacearum* KK5, *P. corrugata* KK7, *Paenibacillus borealis* KK4, and the symbiotic strain *Sinorhizobium meliloti* KK13 affect the expression of genes encoding phenylalanine ammonia-lyase (PAL), the activity of this enzyme, and the production of phenolic compounds in *Medicago truncatula*. Seedlings were inoculated with rhizobacteria, then at T0, T24, T72, and T168 after inoculation, the leaves and roots were analyzed for gene expression, enzyme activity, and the content of phenolic compounds. All bacteria affected *PAL* gene expression, in particular, *MtPAL2*, *MtPAL3*, and *MtPAL4*. *Pseudomonas* strains had the greatest impact on gene expression. The inoculation affected PAL activity causing it to increase or decrease. The most stimulating effect on enzyme activity was observed 168 h after inoculation. A varied effect was also observed in the case of the content of phenolic compounds. The greatest changes were observed 24 h after inoculation, especially with the KK7 strain. The influence of the studied rhizobacteria on the biosynthesis of phenolic compounds at the molecular level (expression of *MtPAL* genes) and biochemical level (PAL activity and content of phenolic compounds) was confirmed. The *MtPAL3* gene underwent the most significant changes after inoculation and can be used as a marker to assess the interaction between *M. truncatula* and rhizobacteria. The *Pseudomonas* strains had the greatest influence on the biosynthesis pathway of phenolic compounds.

## 1. Introduction

Plants live in a complex and dynamic microbial environment [1,2]. The microbiome (microbiota and their genomes) inhabits the soil, the rhizosphere, roots, and all plant tissues. So, it is no surprise that microorganisms play a key role in soil health, nutrient cycling, and plant growth and development. They also modulate the plant’s response to biotic and abiotic environments [2,3,4,5]. Plant–microbe interactions can be beneficial, neutral, or harmful, depending on the specific microbes involved and the context of the interaction [6,7].

Harmful interactions between plants and microbes can have serious consequences for plant and crop health. Pathogenic microbes can cause plant diseases, leading to stunted growth, reduced yields, and even death [8]. However, there are also many others that are beneficial for plants, like rhizobacteria (PGPRs: plant growth-promoting bacteria; PGPRs: plant growth-promoting rhizobacteria), which can improve plant growth and benefit the adaptation of plants to adverse conditions [9,10]. These bacteria can be used as biofertilizers (increase the availability of nutrients for plants), biostimulators (produce hormones or modify their levels in plants), and biopesticides (fight diseases mainly by producing antibiotics, antifungal metabolites, and fungal cell lysis enzymes and inducing systemic resistance) depending on their modes of action [10,11,12]. Bacteria can use single mechanisms to promote plant growth, but usually, multiple mechanisms are activated simultaneously [13]. Inoculation with beneficial microbes can improve plant growth and development, enhance nutrient uptake, and increase disease resistance [14]. For example, inoculation with plant growth-promoting rhizobacteria (PGPR) has been shown to improve the growth and yield of a wide range of crops, including wheat, soybean, maize, and others [11,15]. Inoculation with microbes can also help plants tolerate abiotic stress, such as drought or high salinity [16].

Plants respond to stress signals, both abiotic and biotic (including growth-promoting and plant-pathogenic microorganisms). Some of these reactions are local, while others are systemic in nature, trigger plant-wide defenses, and may lead to induced resistance. Depending on the type of elicitor, we distinguish two basic types of systemic resistance, SAR (Systemic Acquired Resistance), triggered by plant pathogens, and ISR, triggered by mutualistic microorganisms that colonize roots [14,17,18,19,20,21]. PGPRs induce early plant ISR events, including, but not limited to, increased expression of PR genes related to pathogenesis, increased activity of defense-related substances such as polyphenol oxidase, peroxidase, β-1,3-glucanase, and chitinase, and accumulation of reactive oxygen species as well as phenylalanine ammonia-lyase [22,23,24,25]. Phenylalanine ammonia-lyase (PAL) is the first and one of the key enzymes in the biosynthesis of phenolic compounds. PAL catalyzes the non-oxidative deamination of phenylalanine to trans-cinnamic acid and ammonia. Trans-cinnamic acid, in turn, is a common precursor of lignin, and the biosynthesis pathway of flavonoids, the phenylpropanoid pathway, is one of the most important pathways of plant metabolism and is responsible for the production of many secondary metabolites [26,27,28,29,30]. PAL activity depends on the developmental stage, the degree of differentiation of cells and tissues, and exposure to various stress stimuli. In addition, there are many reports of PAL stimulation by infection, mechanical trauma, UV radiation, drought, and temperature changes, as well as induction with methyl jasmonate or methyl salicylate [31]. Increased PAL activity has been correlated with increased production of phenylpropanoid compounds. These compounds, in turn, play defensive or signaling roles in the plant, including antibacterial activity and the synthesis of signaling compounds such as salicylic acid [31]. PAL occurs in all higher plants as well as in yeasts and fungi [32]. It is usually encoded by a small polygenic family of two to five members [33,34]. Four PAL isoforms have been detected in *Arabidopsis thaliana* L. [35,36], five in *Populus* [37], thirteen in *Cucumis sativus* L. [38] and *Cucumis melo* L., sixteen in *Vitis vinifera* L. [39], seven in *Medicago sativa* L. [40], and six in *Medicago truncatula* Gaertn [41].

The use of *Medicago truncatula* as a model organism offers several advantages for studying plant–microbe interactions and the phenylpropanoid pathway. Its short life cycle and ability to form symbiotic relationships with nitrogen-fixing bacteria make it a valuable tool for understanding the complex interactions between plants and microbes [42,43]. Additionally, the *Medicago truncatula* genome has been sequenced and is well annotated, making it a valuable resource for functional genomics and comparative analysis [44].

Understanding the interactions between plants and microbes is therefore crucial for improving plant health and disease resistance, as well as for developing sustainable agricultural practices. By studying the specific mechanisms of plant–microbial interactions, scientists can identify potential intervention targets and develop new strategies to improve yields and reduce the use of synthetic fertilizers and pesticides [45].

The study was aimed at examining the effect of inoculation with free-living and symbiotic rhizobacteria on the biosynthesis of phenolic compounds in *Medicago truncatula* plants, with particular emphasis on the expression of genes encoding *PAL*, PAL enzyme activity, and the total content of phenolic compounds in leaves and roots. It was hypothesized that inoculation with different strains of rhizobacteria would affect the pathway of biosynthesis of phenolic compounds, starting from the expression of key genes in this pathway, through enzymatic activity, up to the total content of phenolic compounds. Although there is knowledge about the response of the phenylpropanoid pathway in the context of biotic and abiotic stress, there is still limited knowledge about the mechanisms involved in the interaction between plants and rhizobacteria, in particular regarding the comprehensive approach to this interaction at the molecular and biochemical level. The present study aimed to fill this knowledge gap and provide insight into the potential impact of free-living and symbiotic rhizobacteria on the phenylpropanoid pathway.

## 2. Results

### 2.1. Effect of Rhizobacteria on the Expression of Phenylalanine Ammonia-Lyase (PAL) Genes

**Figure 1:**
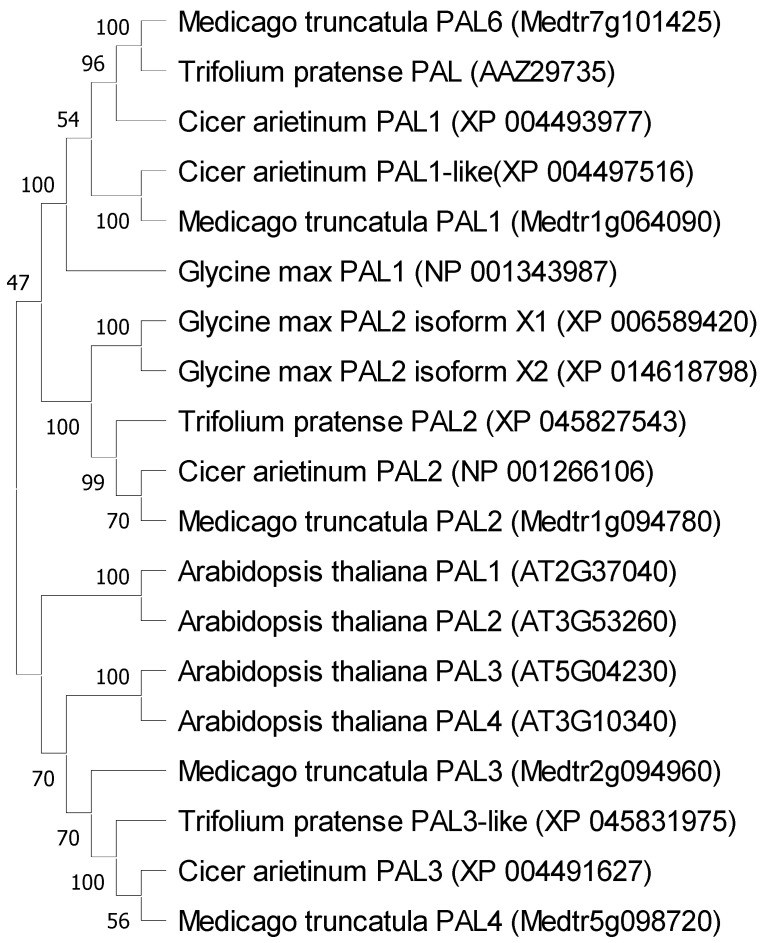
Phylogenetic analysis annotated PALs in *Medicago truncatula*, *Arabidopsis thaliana*, and other species of plants belonging to the *Fabaceae* family, created on the basis of amino acid sequences using the Neighbor-Joining method.

The genes encoding phenylalanine ammonia-lyase from *M. truncatula* were shown to be similar to genes encoding this enzyme in other plants. It should be noted that the description of the affiliation of individual PALs to the appropriate classes requires unification. The *M. truncatula* amino acid sequences for PAL1 and PAL6 were similar and belonged to one clade. PAL 2 was in the next clade, and PAL3 and PAL4 were in the last one (Figure 1).

In the roots and leaves of both inoculated and non-inoculated plants, expression of all five *MtPAL* genes was found, with the expression of *MtPAL3* and *MtPAL4* genes becoming visible a little later. In non-inoculated seedlings of *M. truncatula*, higher expressions of *MtPAL1*, *MtPAL2*, *MtPAL3*, and *MtPAL4* genes were observed in the roots, while the *MtPAL6* gene was expressed at a similar level in leaves and roots (Figure S1). The expression of all tested *MtPAL* genes in the leaves of 5-week-old *M. truncatula* seedlings changed after inoculation with bacteria. The increase in expression of these genes was especially evident after 72 h (Figure S1). Also, in the roots of inoculated seedlings, changes in the expression of genes encoding phenylalanine ammonia-lyase were observed, often visible after 24 h (Figure S1). At the same time, it was observed that the expression of the two genes, *MtPAL1* and *MtPAL6*, is constantly at a fairly high level, which may indicate that these genes are leading in the biosynthesis of phenolic compounds. Although they also reacted to inoculation with bacteria, it was to a small extent. Out of five genes for quantitative qPCR analysis, *MtPAL2*, *MtPAL3*, and *MtPAL4* were selected due to the observed change in their expression profile in leaves and roots of *M. truncatula* seedlings inoculated with bacteria in relation to untreated plants. They can be markers of induced systemic resistance.

**Figure 2:**
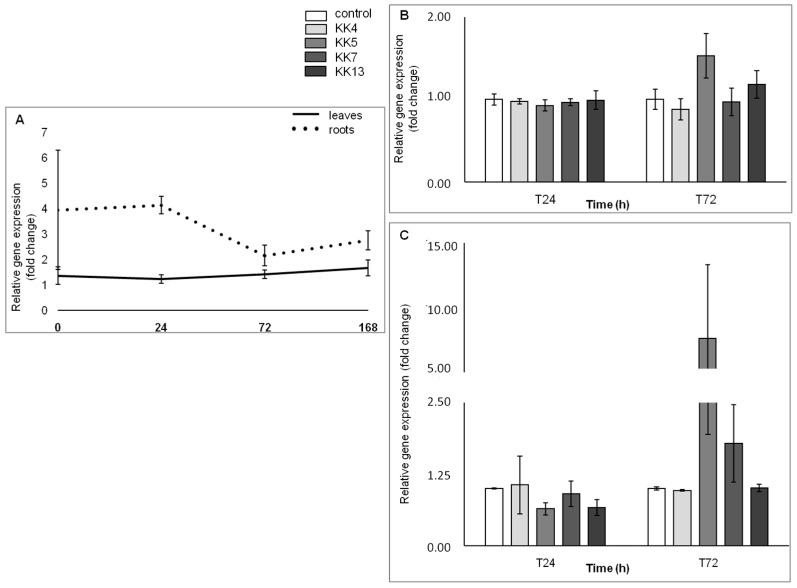
*MtPAL2* gene expression profile in leaves and roots of non-inoculated *M. truncatula* seedlings (at time points: T0—seedlings aged 35 days; T24—36 days; T72—38 days; T168—42 days) (**A**) and the effect of rhizobacteria on the expression of this gene in leaves (**B**) and roots (**C**) 24 and 72 h after inoculation of 35-day-old seedlings.

**Figure 3:**
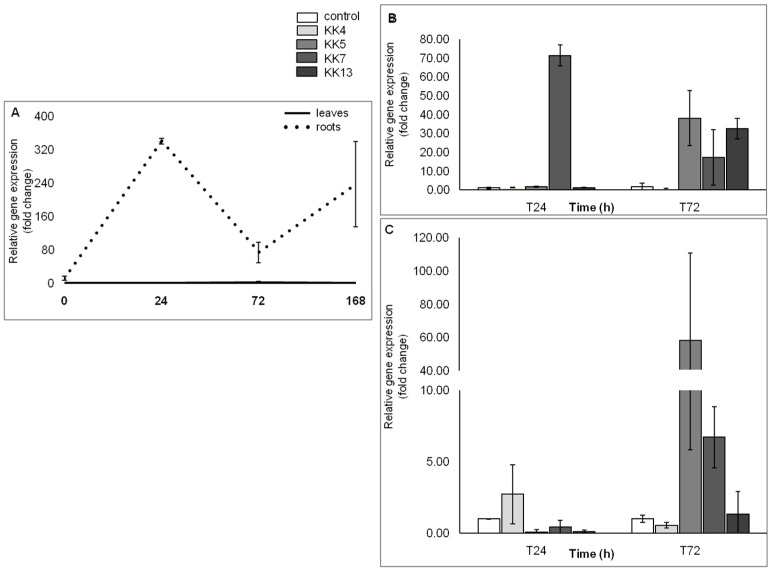
*MtPAL3* gene expression profile in leaves and roots of non-inoculated *M. truncatula* seedlings (at time points: T0—seedlings aged 35 days; T24—36 days; T72—38 days; T168—42 days) (**A**) and the effect of rhizobacteria on the expression of this gene in leaves (**B**) and roots (**C**) 24 and 72 h after inoculation of 35-day-old seedlings.

**Figure 4:**
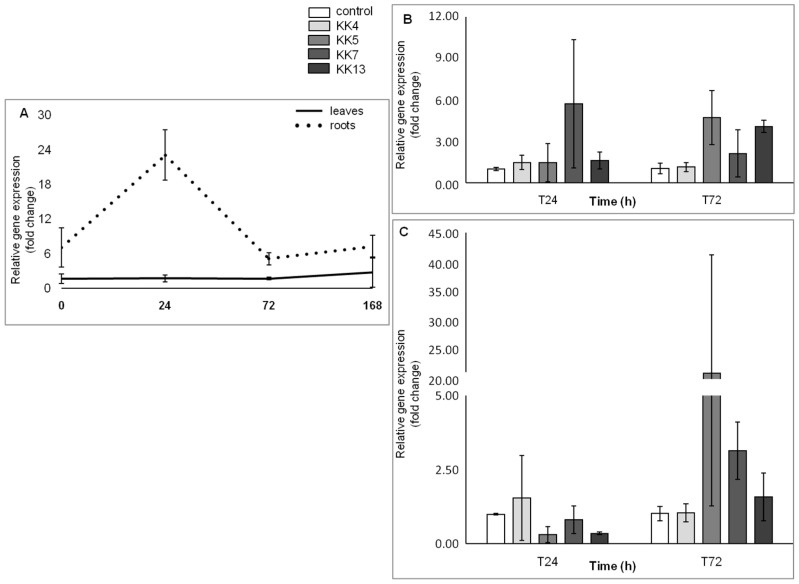
*MtPAL4* gene expression profile in leaves and roots of non-inoculated *M. truncatula* seedlings (at time points: T0—seedlings aged 35 days; T24—36 days; T72—38 days; T168—42 days) (**A**) and the effect of rhizobacteria on the expression of this gene in leaves (**B**) and roots (**C**) 24 and 72 h after inoculation of 35-day-old seedlings.

**Figure 5:**
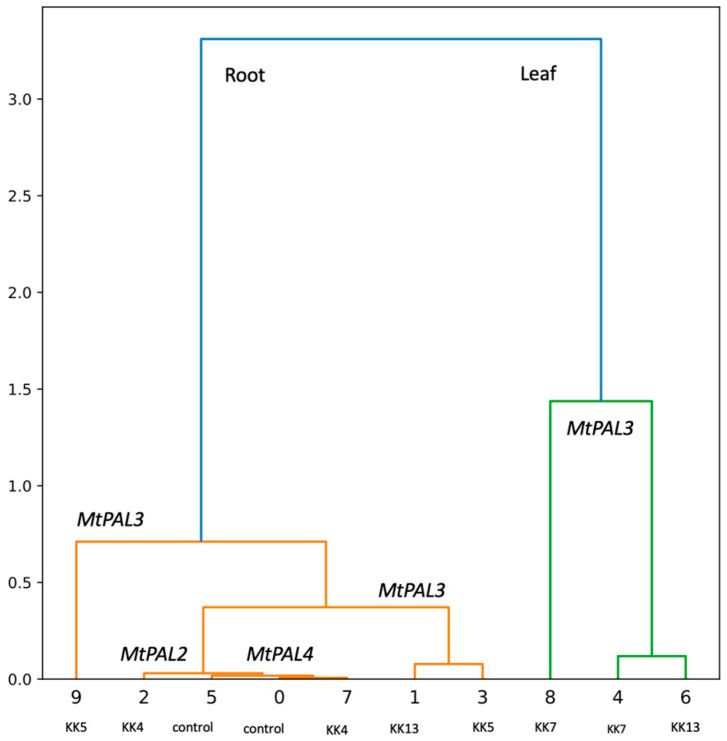
Hierarchical cluster analysis showing the effect of rhizobacteria on *MtPAL* gene expression after *M. truncatula* inoculation.

The results obtained with the qPCR method confirmed those previously obtained with the semi-quantitative PCR method and indicated that the expression level of *MtPAL2*, *MtPAL4*, and *MtPAL3* genes in the roots of non-inoculated plants is higher than in the leaves (Figure 2A, Figure 3A and Figure 4A), which was also confirmed by hierarchical cluster analysis (Figure 5). The level of *MtPAL2* gene expression in the roots at T0 and T24 was about 4 times higher, and after 168 h, about 1.7 times higher than in leaves (Figure 2A). Inoculation of seedlings with the bacterial suspension had no effect on the level of *MtPAL2* expression in leaves after 24 h (Figure 2B), while after 72 h, a 1.5-fold increase in the level of gene expression was observed under the influence of *Pseudomonas brassicacearum* KK5 (Figure 2B). In the roots, 24 h after inoculation of the seedlings with a suspension of two strains of *P. brassicacearum* KK5 and *Sinorhizobium meliloti* KK13, a significant decrease in *MtPAL2* gene expression was observed or, as in the case of inoculation with other strains, no effect. However, 72 h after inoculation of *P. brassicacearum* KK5 and *P. corrugata* KK7, the amount of *MtPAL2* gene transcript in the roots increased almost 8-fold and 1.8-fold, respectively (Figure 2C). Another *PAL3* gene showed strong root specificity because its expression level in the root at times T0, T24, T72, and T168 was higher than that in the leaf by 12, 340, 74, and 238 times, respectively, while in the leaves, it showed a constant low level (Figure 3A). On the other hand, inoculation of seedlings with a suspension of *Pseudomonas* bacteria i.e., *P. corrugata* KK7 and *P. brassicacearum* KK5, and the symbiotic strain *Sinorhizobium meliloti* KK13 resulted in a significant increase in the expression of the *MtPAL3* gene in the leaves. The *P. corrugata* KK7 strain caused a very large increase in the expression of this gene after 24 h and 72 h, by 71- and 17-fold, respectively, and the inoculation of *P. brassicacearum* KK5 and *Sinorhizobium meliloti* KK13 resulted in an increase in expression, respectively, by 38- and 32-fold after 72 h (Figure 3B). In the roots, a decrease in *MtPAL3* gene expression was observed 24 h after inoculation of *P. brassicacearum* KK5, *P. corrugata* KK7, and *S. meliloti* KK13, followed by an increase 72 h after inoculation of *P. brassicacearum* KK5 (58 times) and *P. corrugata* KK7 (6.7 times)—Figure 3C.

The *MtPAL4* gene, like *MtPAL2*, showed a higher level of expression in the roots. The expression level of *MtPAL4* in the roots at T0, T24, and T72 was 6-, 23-, and 4-fold higher, respectively, than in leaves. As in the case of the *MtPAL2* gene, the expression of *MtPAL4* in the leaves increased steadily and reached a 2.8-fold increase in 6-week-old plants (168 h) compared to leaves from 5-week-old plants (Figure 4A). In leaves 24 h after inoculation of *Pseudomonas corrugata* KK7, *P. brassicacearum* KK5, and *Sinorhizobium meliloti* KK13, the expression level increased 5.7-, 4.7-, and 4-fold (Figure 4B). In the roots, the amount of transcript of this gene 24 h after inoculation of *P. brassicacearum* KK5 and *S. meliloti* KK13 decreased by about 0.3 times, and after 72 h after inoculation of *P. brassicacearum* KK5 and *P. corrugata* KK7 seedlings, it increased 21 and 3 times, respectively (Figure 4C).

The results of the conducted experiments indicate that the inoculation of *M. truncatula* seedlings with suspensions of selected bacteria caused changes, increases or decreases, in the expression of the three analyzed *MtPAL* genes both in leaves and roots. Of the four strains used for the analysis, i.e., *Paenibacillus borealis* KK4, *Pseudomonas brassicacearum* KK5, *Pseudomonas corrugata* KK7, and *Sinorhizobium meliloti* KK13, only *P. borealis* KK4 had little effect on the expression of the *MtPAL2*, *MtPAL3*, and *MtPAL4* genes in both the roots and leaves of *M. truncatula*. The remaining three strains were able to induce very high expressions of genes, especially *MtPAL3* (Figure 5), which may indicate a systemic response to the inoculation of *M. truncatula* seedlings with these bacteria. It seems that the *MtPAL3* gene may be an indicator for the analysis of interactions between rhizobacteria and *M. truncatula*.

### 2.2. Effect of Rhizobacteria on Phenylalanine Ammonia-Lyase Activity

**Figure 6:**
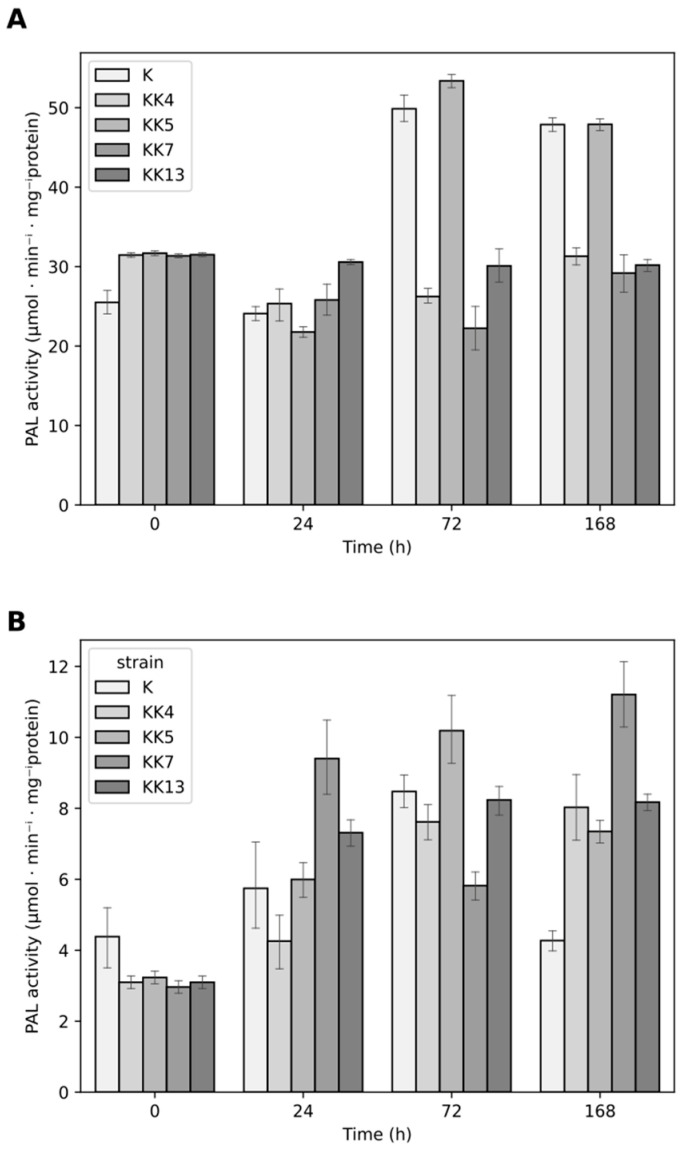
Effect of rhizobacteria on phenylalanine ammonia-lyase (PAL) activity in leaves (**A**) and roots (**B**) of 5-week-old seedlings of *M. truncatula* at 24, 72, and 168 h after inoculation.

Phenylalanine ammonia-lyase (PAL) is one of the first enzymes in the pathway of biosynthesis of phenolic compounds. It converts phenylalanine to cinnamic acid and is one of the markers of plant systemic resistance. The activity of this enzyme was checked in the leaves and roots of 5-week-old *M. truncatula* seedlings just before and 24, 72, and 168 h after inoculation with rhizobacteria (Figure 6). The activity of the PAL enzyme was approximately 5-fold higher in the leaves compared to the roots, and in addition, the activity in the leaves, which resulted from the systemic response after inoculation of the roots with rhizobacteria, was very stable. A 2-fold increase in the activity of this enzyme was observed in the leaves of non-inoculated plants that were 38 days old (72 h), which was maintained for the next 4 days (168 h)—Figure 6A. In the roots of non-inoculated 38-day-old plants, an increase in the activity of this enzyme was observed by 47%, and in the 6-week-old plants, it decreased by 25% (Figure 6A,B).

**Figure 7:**
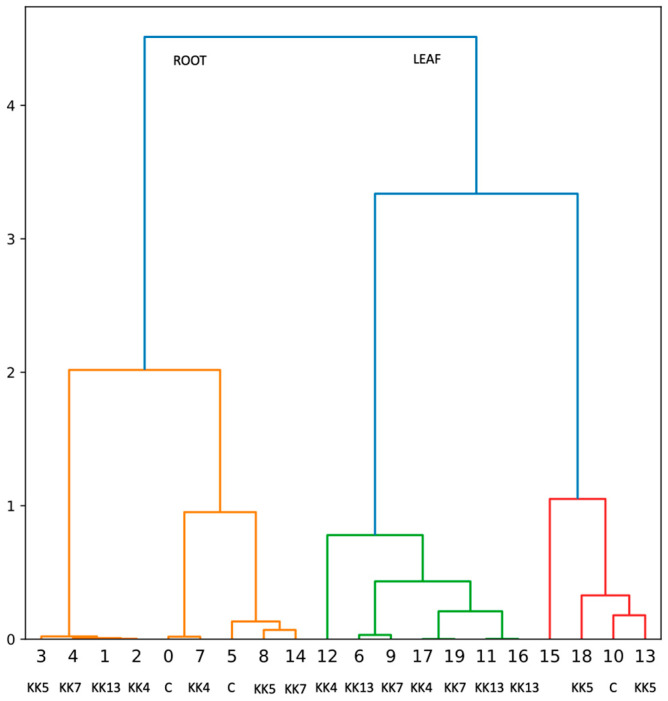
Hierarchical cluster analysis showing the effect of rhizobacteria on PAL enzyme activity after *M. truncatula* inoculation.

Changes in the activity of this enzyme in leaves and roots after treatment with rhizobacteria were visible (Figure 6). Slight changes were observed in the leaves 24 h after inoculation, with three strains inducing PAL activity: most effectively *Sinorhizobium meliloti* KK13—27%; then *Pseudomonas corrugata* KK7—7.1%; and *Paenibacillus borealis* KK4—5.2%. Three days after inoculation, only one strain, *P. brassicacearum* KK5, slightly increased PAL activity by 7%, while the others decreased it by 39 to 55%, and after another 4 days, most of the strains significantly decreased enzyme activity (from 35 to 39%). It should be noted, however, that taking into account the dynamics of changes, the activity of the PAL enzyme in the leaves of plants treated with three strains (KK4, KK7, and KK13) remained at a similar level, while inoculation with *P. brassicacearum* KK5 increased the activity between days 1 and 3 by 145% and reached a level close to the control (Figure 6A and Figure 7).

In roots, PAL activity increased 24 h after inoculation with *P. corrugata* KK7 and *S. meliloti* KK13, by 9.4 and 7.3%, respectively. After 72 h, the *P. brassicacearum* KK5 strain induced resistance by 28.4%, while *P. corrugata* KK7 inhibited PAL activity by 31.3%. Inoculation with all strains increased PAL enzyme activity in roots 168 h after inoculation. In particular, *P. corrugata* KK7 increased activity by 162%, followed by *S. meliloti* KK13 by 93%, *P. borealis* KK4 by 88%, and *P. brassicacearum* KK5 by 72% (Figure 6B). The effect of these strains on PAL activity in the roots was also confirmed by hierarchical cluster analysis (Figure 7).

### 2.3. Effect on the Content of Phenolic Compounds

**Figure 8:**
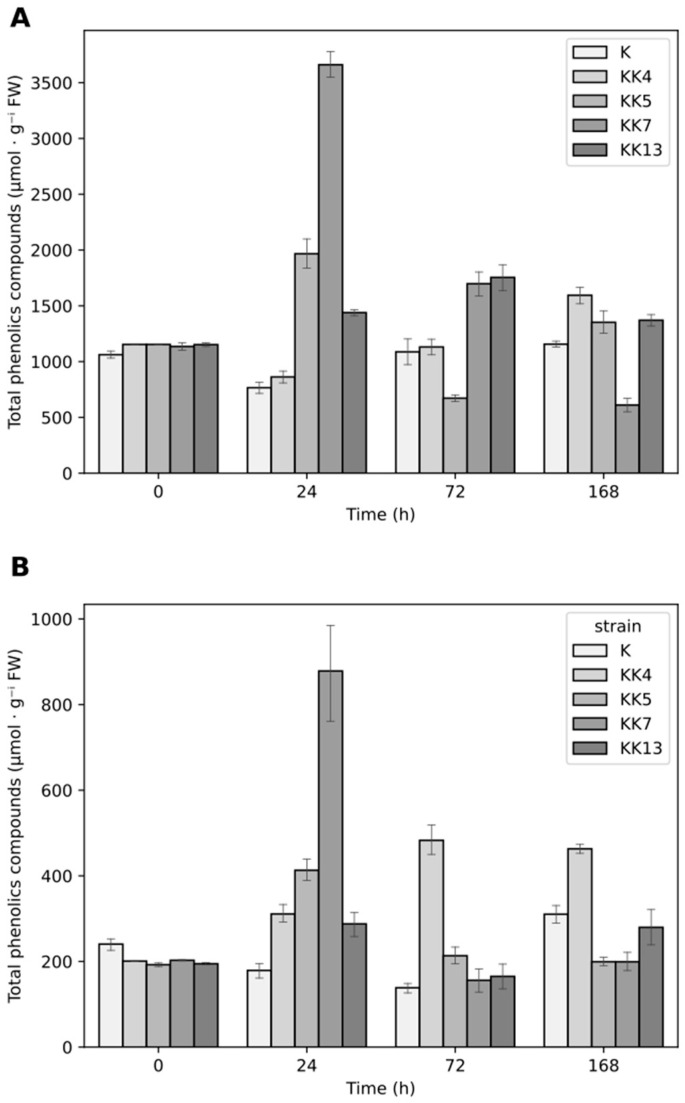
Effect of rhizobacteria on the content of phenolic compounds in leaves (**A**) and roots (**B**) in 5-week-old *M. truncatula* seedlings at 24, 72, and 168 h after inoculation.

**Figure 9:**
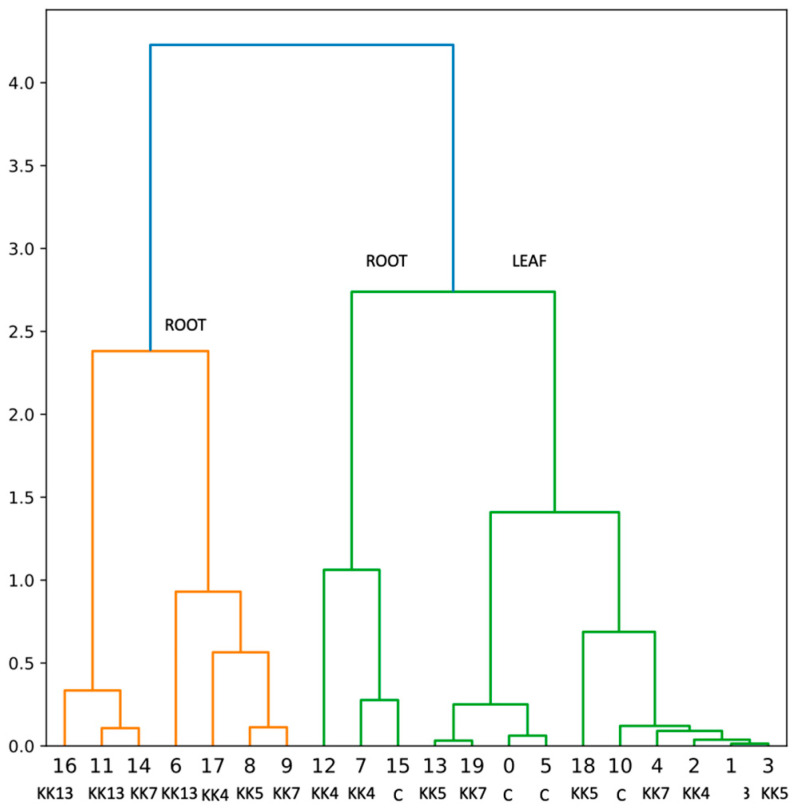
Hierarchical cluster analysis showing the effect of rhizobacteria on total phenolic content after *M. truncatula* inoculation.

Five-week-old leaves of *M. truncatula* control seedlings contained four times more phenolic compounds than the roots of these seedlings (Figure 8 and Figure 9), which was therefore consistent with the previously described PAL activity.

After inoculation with bacteria, changes in the total content of phenolic compounds were observed in the leaves and roots of the seedlings compared to the control (Figure 8). In the leaves 24 h after the inoculation of *Pseudomonas corrugata* KK7, *P. brassicacearum* KK5, and *Sinorhizobium meliloti* KK13, an increase in the content of phenolic compounds by 378, 157, and 88% was observed, respectively. After 72 h, an increase in the content of phenols was visible after treatment with the suspension of *P. corrugata* KK7 by 93% and *S. meliloti* KK13 by 99%. The increase in the content of these compounds in the leaves by 38% was also caused by the inoculation of *Paenibacillus borealis* KK4 after 168 h, while the inoculation of *Pseudomonas corrugata* KK7 reduced the content of phenolic compounds in the leaves (Figure 8A). Inoculation of seedlings with a suspension of *P. brassicacearum* KK5 and *P. corrugata* KK7 resulted in an increase in the content of phenols after 24 h by 130 and 390%, respectively, and *Paenibacillus borealis* KK4 increased after 72 and 168 h by 267 and 49%, respectively (Figure 8B).

## 3. Discussion

Phenolic compounds play a key role in plant interactions with the environment, including microorganisms. Due to their high biological activity and the ability to be secreted from root tissues into the environment, they are the first line of defense against pathogens. The first enzyme in the biosynthesis of the phenylpropanoid pathway is phenylalanine ammonia-lyase—PAL [34]. The products of this pathway are soluble phenols, flavonoids, and lignins, which are key factors in plant disease resistance [41]. PAL is also a key enzyme in the biosynthesis of salicylic acid (SA), which is responsible for triggering systemic acquired immunity (SAR) in plants [46]. PAL gene expression in plants can be increased by various environmental factors including pathogen infection, insect bites, UV radiation, and extreme temperatures [26,47]. In addition, it is believed that the production of phenolic compounds and the activity of phenylalanine ammonia-lyase (PAL) can serve as markers for the induction of systemic immunity [26,31,48,49].

Therefore, it was analyzed whether the expression of genes encoding PAL, the activity of this enzyme, and the content of its products, i.e., phenols, change after 1, 3, and 7 days after inoculation with selected strains of rhizobacteria in the roots and leaves of 5-week-old *M. truncatula* seedlings. The influence of free-living and symbiotic rhizobacteria on the biosynthesis pathway of phenolic compounds of *M. truncatula* plants at the molecular and biochemical levels was confirmed [41,46,48].

In the genome of Medicago truncatula, six genes encoding phenylalanine ammonia-lyase were found [41], which is two more than in *Arabidopsis thaliana*—four *PAL* genes [47]—and three less than in *Oryza sativa*—nine *PAL* genes [50]. The similarity of five genes from *M. truncatula* to homologous genes from other plants of the *Fabaceae* family was also confirmed (Figure 1) such as *Trifolium pratense*, *Cicer arietinum*, and *Glycine max*. It was observed that the *MtPAL1*, *MtPAL2*, *MtPAL3*, and *MtPAL4* genes show higher expressions in the roots, while the *MtPAL6* gene expression was at a similar level in the leaves and roots of 35–42-day-old *M. truncatula* J5 seedlings (Figure S1, Figure 2 and Figure 4). In turn, Ren et al. (2019) [41] tested *M. truncatula* A17 seedlings during flowering using a microarray and they found that the roots had a higher expression of *MtPAL2*, *MtPAL1*, and *MtPAL6* genes in the leaves, and the expression of *MtPAL4* was visible at a similar level in these organs. However, it should be remembered that a different line of *M. truncatula* at a different age was analyzed for the presented study. The response of *PAL* genes to drought stress and salinity in *M. truncatula* was demonstrated. Their role in response to environmental stress has also been proven in *Arabidopsis*. In addition, the knockout of four genes encoding *PAL* resulted in susceptibility to *Pseudomonas syringae* [47]. Similarly, the PAL genes from *Medicago sativa* have been shown to respond to salinity [40]. The other studies have demonstrated the induction of *PAL* genes by pathogens [27,51,52,53]. However, there are not many reports on the induction of *PAL* genes in plants after inoculation with rhizobacteria. All five *MtPAL* genes tested in *M. truncatula* were upregulated after inoculation with rhizobacteria, although the increase in expression of three (*MtPAL2*, *MtPAL3*, and *MtPAL4*) was particularly noticeable. The other two genes (*MtPAL1* and *MtPAL6*) were characterized by a relatively constant, high level of expression in the observed time interval between 35 and 42 days of plant development and were slightly affected by bacterial inoculation (Figure S1). Therefore, it can be considered that these are the lead genes responsible for maintaining the production of phenolic compounds in *M. truncatula*. A significant effect of inoculation of seedlings with rhizobacteria on the increase in expression of *MtPAL2*, *MtPAL3*, and *MtPAL4* genes in roots was confirmed, especially after treatment of seedlings with strains belonging to *Pseudomonas* (*P. brassicacearum* KK5 and *P. corrugata* KK7)—Figure 2, Figure 3, Figure 4 and Figure 5. The greatest induction of expression after inoculation with rhizobacteria was observed for the *MtPAL3* gene, which also showed strong root specificity; however, induction of expression was also seen in leaves (Figure 4 and Figure 5). The increase in the expression level of this gene in the roots 72 h after inoculation with *Pseudomonas brassicacearum* KK5 strain was 58-fold (Figure 4). The *MtPAL3* gene can therefore be considered the best marker of *M. truncatula* interaction with free-living rhizobacteria. Previously, one of the rice *PAL* genes was observed to be differentially expressed in rice roots during association with *Azospirillum brasilense* [54]. The expression pattern of these flavonoid synthesis genes further suggests that flavonoids likely play a key role in the beneficial associations of rice with bacteria. S. Jain [55] found induction of the *pal2* gene from *Glycine max* (which is in the same clade as *MtPAL2*—Figure 1) after inoculation with *Bacillus* sp. rhizobacteria. They noted the greatest increase in gene expression after *Bacillus* sp. and *Fusarium oxysporum* co-inoculation. In addition, this was higher than pathogen-only inoculation. This may indicate that earlier induction with rhizobacteria increases the response against pathogens and may be related to the induction of resistance in plants. In *Lotus japonicus*, the *PAL* gene (*LjPAL1*) has been shown to be induced by *Mesorhizobium loti* infection and has been found to influence rhizobium infection progression and nodule structure [56]. The *MtPAL1* gene was also tested after inoculation with *Sinorhizobium meliloti* and a minimal reduction in expression was observed in the roots of *Medicago truncatula*, which decreased more after pathogen treatment. They concluded that rhizobacteria inhibit the immune response caused by bacterial pathogens [57]. We also did not observe an increase in expression of this gene after inoculation of *S. meliloti* (Figure S1). These and previous studies indicate the functional diversity of this family of genes.

Increased expression of *MtPAL* genes (mainly *MtPAL3*) by some strains, i.e., *P. brassicacearum* KK5 and *P. corrugata* KK7, was accompanied by increased activity of PAL enzymes and production of phenols, compounds known to have antifungal activity (Figure 6, Figure 7, Figure 8 and Figure 9). However, it is noteworthy that the enzymatic activity in the leaves is higher than in the root, which does not correlate with the results of expression of the *MtPAL2*, *MtPAL3*, and *MtPAL4* genes (Figure 2, Figure 3 and Figure 4). This may be related to the high levels of expression of the leading genes *MtPAL1* and *MtPAL6* (Figure S1) in the leaves observed in the semi-quantitative analysis (expression of these genes was not quantified), as well as post-translational changes. Ali and McNear (2014) [58] showed an increase in the content of flavonoids, as well as a significant activation of genes for the biosynthesis of phenolic compounds (such as chalcone synthase and isomerase, flavonoid hydroxylase, or flavonol synthase) in *Arabidopsis thaliana* leaves after treatment with Soil-Builders AF microbiological preparation containing rhizobacteria, with no induction of expression for all four *PAL* genes. Inoculation of rice with thirty isolates of rhizobacteria (most of which belonged to the genus *Bacillus*, then to *Staphylococcus*) showed that most of the isolates resulted in an increase in PAL enzyme activity, with 86.6% in shoots and 53.3% in roots [59]. An increase in PAL activity was also observed after wheat inoculation with *Stenotrophomonas maltophilia* rhizobacteria. The further increase in PAL activity was influenced by the subsequent contact of the plant with the pathogen [60]. However, after inoculation by means of microinjections and spraying of leaves of 4-week-old chickpea seedlings with both *Pseudomonas fluorescens* and *P. aeruginosa* strains, an increase in PAL activity was noted after 1, 2, and 3 days after the treatment, with the largest approx. increase for the control observed after the first day, and the maximum activity of the PAL enzyme observed after 2 days [61]. Subsequently, in studies conducted on chickpeas, a higher collection of phenolics was observed 1 to 3 days after treatment of four-week-old seedlings with PGPR strains (especially in the presence of the pathogen—*Sclerotinia sclerotinum*), which was attributed to the activation of the ISR mechanism by bacterial strains [61]. An increase in the content of phenolic acids and the total content of phenols was also noted by Bahadur et al. (2007) [62] 48 and 96 h after inoculation of pea leaves with strains belonging to *Pseudomonas fluorescens* or *Pseudomonas aeruginosa*. The same strains used to inoculate chickpea seeds caused an increase in the content of phenolic compounds in 4-week-old leaves of seedlings, and this increase was especially significant when both strains were used together. In addition, the authors found a correlation between the accumulation of phenols and the protection of chickpeas against infection by the fungus *Sclerotinum ralstoni* [63]. There was also an accumulation in the roots in the first week after inoculation of salicylic acid, a key phenolic compound playing an important role in signaling during immunity induction, with a complete lack of this acid in the rest of the plant. These authors believed that the salicylic acid accumulated in the roots is of bacterial origin [63]. An increase in PAL activity and phenol content was also observed in pea and chickpea leaves after infection with the pathogen *Sclerotina sclerotinum* after prior inoculation with a mixture of microorganisms (microbial consortium) consisting of bacteria belonging to the genus *Pseudomonas* and *Bacillus* and the fungus *Trichoderma harzianum* [64,65]. An increase in the activity of the PAL enzyme and the content of phenolic compounds was also observed in rice after inoculation with *Pseudomonas fluorescens* [66]. A similar relationship, i.e., a four-fold increase in the total content of phenolic compounds, was observed in leaves and roots 24 h after inoculation of *M. truncatula* seedlings with the *Pseudomonas corrugata* KK7 strain (Figure 8). Plant growth-promoting rhizobacteria are known to increase resistance to abiotic and biotic stress, including by activating the phenylpropanoid pathway. This can lead to the emergence of plant resistance and the control of plant diseases. However, before rhizobacteria can be used to improve crop yields, it is essential to understand the mechanisms behind plant–microbe interactions. The study shows the potential of rhizobacteria to stimulate the phenylpropanoid pathway. The next step to complete the study would be to see if rhizobacteria also trigger a response in the expression of the *MtPAL5* gene, which has not been studied, and other genes and enzymes in this pathway, such as chalcone synthase and chalcone isomerase. It would also be worth analyzing changes in the path under study after contact with the pathogen.

## 4. Materials and Methods

### 4.1. Plant Culture and Inoculation

*Medicago truncatula* Gaertn. ecotype Jemalong 5 was used in the research (French National Institute for Agricultural Research—INRAE, Paris, France). After chemical scarification and stratification at 4 °C, the seeds were transferred to pots containing a mixture of perlite and sand in a 1:1 ratio (*v*/*v*). *Medicago* seedlings were watered with a low-nitrogen medium containing NPK-18:6:26 mineral compounds. The plants were grown at a light intensity of 150 μmoL·m^−1^·s^−1^, a photoperiod of 16 h/8 h, and a temperature of 24 °C/20 °C [67].

*Pseudomonas* rhizobacterial strains (*P. brassicacearum* KK5 and *P. corrugata* KK7), *Paenibacillus borealis* KK4, and the symbiotic strain *Sinorhizobium meliloti* KK13 were used. Bacterial cultures were grown in a Tryptic Soya Broth medium (Scharlau, Barcelona, Spain) until OD_600_ was about 0.6. The cultures were then centrifuged (4 °C/5000 rpm/20 min). The supernatant was carefully removed, and the bacterial cell pellet was resuspended in the same volume of 10 mM magnesium sulfate solution. The procedure was repeated twice. The bacterial suspension prepared in this way was diluted with 10 mM magnesium sulfate solution to a density of 10^8^ CFU mL ^−1^ (0.1 optical density) and was used for inoculating 5-week-old seedlings of *Medicago truncatula* J5. For this purpose, 10 mL of freshly prepared bacterial inoculum was pipetted near the root system on the substrate at a distance of 1 cm from the shoot. Control seedlings were treated with a 10 mM magnesium sulfate solution. Material for analysis was collected just before inoculation (T0), 24 h after inoculation (T24), 72 h after inoculation (T72), and 168 h after inoculation (T168).

### 4.2. Determination of Total Phenolic Compounds

The content of phenolic compounds was determined by the Folin–Ciocalteau method using a UV-1800 spectrophotometer (Shimadzu, Duisburg, Germany). Briefly, 0.1 g of leaf and root tissue was homogenized in 1 mL of 80% (*v*/*v*) ethanol. The reaction mixture was prepared with 0.5 mL of supernatant, 0.08 mL of 20% (*w*/*v*) NaCO_3_ in water, and 0.24 mL of Folin–Ciocalteau reagent. After 60 min, the absorbance was measured at 760 nm. The total phenol content was calculated from the calibration curve prepared for chlorogenic acid, and the content of phenolic compounds was expressed as mmoL of chlorogenic acid/g of fresh weight.

### 4.3. Analysis of Phenylalanine Ammonia-Lyase (PAL) Activity

PAL activity was determined by a spectrophotometric method [68], based on the transformation of phenylalanine into trans-cinnamic acid at 290 nm. In total, 200 mg of leaf or root tissue was homogenized in liquid nitrogen, then added to 2 mL of extraction buffer containing 50 mM Tris-HCl pH 8.8 and 15 mM β-mercaptoethanol (Scharlau, Spain), and centrifuged for 15 min at 16,000× *g* at 4 °C. The supernatant was used for enzyme activity assays and protein content determination. The reaction mixture was prepared in a spectrophotometer cuvette with 100 µL of extraction buffer, 50 µL of 10 mM phenylalanine (Scharlau), 30 µL of deionized water, and 20 µL of plant extract, and incubated at 37 °C for 1 h. After that, the reaction was stopped by adding 50 µL of 6 M HCl, and the absorbance was measured at 290 nm. Enzyme activity was calculated from the standard curve in the range of 20–200 nmoL of trans-cinnamic acid and expressed as µmoL of trans-cinnamic acid/mg of protein. Protein content was measured using the Bradford method (1976) [69].

### 4.4. Effect of Bacteria on PAL Gene Expression in M. truncatula

#### 4.4.1. RNA Isolation from Roots/Seedlings and Reverse Transcription

Approximately 200 mg of plant tissue from leaves or roots of *M. truncatula* seedlings grown in pots or roots of seedlings grown in plates was ground in liquid nitrogen using a mortar and pestle. RNA isolation was carried out using a Direct-zol RNA isolation kit (Zymo Research, Irvine, CA, USA). The isolated RNA was used for further procedures or stored at −80 °C. RNA concentration and purity were assessed by spectrophotometric measurement (BioSpec Nano, Schimadzu, Kyoto, Japan). cDNA synthesis was performed using 500 ng of isolated mRNA and the GoScript Reverse Transcription System (Promega, Madison, WI, USA). The resulting cDNA was stored at −20 °C and used for analysis after 5-fold dilution.

#### 4.4.2. Sequence Alignment and Primer Design for Gene Expression Analysis

The gene sequences available in the J. Craig Venter database (http://www.jcvi.org/medicago, accessed on 20 February 2021) were used for designing primers for the target genes and reference genes. Sequence alignments of PAL proteins from *M. truncatula*, *Arabidopsis thaliana* from Tair (The Arabidopsis Information Resource) Database [36], and other *Fabaceae* species (NCBI Database) were performed using ClustalX2 conducted on MEGA software (Molecular Ewolutionary Genetics Analysis, X version, Masatoshi Nei, Richlandtown, PA, USA). The sequence alignment was used to construct a phylogenetic tree using the Neighbor-Joining method, and bootstrap analysis of 1000 replicates was used to assess the confidence level of monophyletic groups. All sequences used are listed in Table 1. The reference gene was selected based on previous reports of gene expression studies in similar systems of *Medicago truncatula*. Jasiński et al. (2009) [67] also used actin 2 as a reference gene and examined the effects of microorganisms, symbiotic bacterium *Sinorhizobium meliloti*, and fungal pathogens, *F. culmorum* and *P. medicaginis* on *M. truncatula* J5 seedlings.

PCR primers for both semi-quantitative and quantitative methods were designed using Primer Express 3.0 software (Applied Biosystems, Carlsbad, CA, USA)—Table 1. Strict primer design conditions were applied. The qPCR primers amplified a fragment of approximately 90 bp within the 400 bp fragment amplified in the semi-quantitative PCR reaction. Additionally, all designed primers had a melting temperature of 58–60 °C.

#### 4.4.3. Semi-Quantitative PCR Analysis of Gene Expression

PCR amplification of fragments of the analyzed genes was performed using the Biometra T Gradient thermocycler. For this purpose, a reaction mixture was prepared containing cDNA and Promega’s GoTaq G2 Hot Start Master Mix. The reaction mixture of 25 µL consisted of 12.5 µL Master Mix (which included 1 U of G2 Hot Start polymerase, 2× reaction buffer pH 8.5, 400 µL of each dNTP, and 4 mM MgCl_2_), 1 µM of each primer, 1 µL cDNA, and nuclease-free water. The polymerase chain reaction was carried out using a three-step protocol. The initial denaturation was 96 °C/2 min; denaturation 96 °C/15 s, primer annealing 60 °C/15 s, and extension 72 °C/45 s repeated for 30 cycles; and final extension 72 °C/5 min. The obtained PCR products of approximately 400 bp were subjected to electrophoresis on a 1.5% agarose gel with ethidium bromide at 70 V for about 40 min using the Bio-Rad electrophoresis apparatus. Each well was loaded with 12 µL of a mixture consisting of 2 µL of PCR product, 2 µL of 6× concentrated loading buffer, and 8 µL of water. A DNA ladder marker (Mass Ruler DNA Ladders 100 bp, Fermentas, Burlington, ON, Canada) was loaded into one of the wells. For the qPCR analysis, genes were selected that were shown to visibly respond to treatment with rhizobacteria in the PCR reaction. Real-time PCR was performed using a two-step protocol with SYBR GREEN dye. cDNA served as a template for amplification in a StepOnePlus™ Real-Time PCR System (Life Technologies, Carlsbad, CA, USA). The reaction mixture included 1 µ of diluted cDNA, 200 nM of each primer, and 2× Master Mix SYBR, in a final volume of 10 µL. The reaction was carried out according to a standard protocol with initial denaturation at 95 °C for 2 min, followed by cycling at 95 °C for 15 s and 60 °C for 60 s, repeated for 40 cycles. Melting curve analysis was then conducted at 95 °C for 15 s, 60 °C for 60 s, and 60–95 °C with an increase of 0.3 °C per cycle. No nonspecific products were detected, and the efficiency of all reactions was 90–95%. The relative expression for each gene was calculated using the 2^−ΔΔCt^ method [70] and normalized to ACT2. Computer analysis was performed using the GenEX 7.0 Std. software (MultiD Analyses AB, Västra Frölunda, Sweden).

### 4.5. Statistical Analyses

To investigate the effects of various factors on the response variable, we employed a comprehensive set of analyses, including (1) a one-way analysis of variance (ANOVA) with interaction terms, followed by the post hoc Tukey Honestly Significant Difference test (HSD), and (2) hierarchical clustering analysis (HCA) using Ward’s linkage method and Euclidean distance metric. Additionally, scatterplots were utilized to provide a visual representation of the relationships within our standardized dataset. All statistical analyses were executed using Python version 3.9.7 in PyCharm Professional 2023 (jetbrains.com). The specific libraries used were pandas, seaborn, statsmodels.api, bioinfokit, scikit-learn, scipy.stats, NumPy, and scipy.cluster.hierarchy. The source code and required materials can be found in a publicly accessible repository on GitHub (https://github.com/PTBDBIODATA, accessed on 10 October 2024).

## 5. Conclusions

The results of these studies provide the first evidence of changes in the biosynthetic pathway of phenolic compounds in model plant *Medicago truncatula* under the influence of free-living and symbiotic rhizobacteria. Plant growth-promoting rhizobacteria strains *Pseudomonas brassicacearum* KK5, *P. corrugata* KK7, and *Paenibacillus borealis* KK4 and the symbiotic strain *Sinorhizobium meliloti* KK13 had a stimulating effect on changes in the expression of genes encoding PAL, in particular, *MtPAL2*, *MtPAL3*, and *MtPAL4*, on the activity of phenylalanine ammonia-lyase and on the total content of phenolic compounds. It was also found that the *MtPAL3* gene reacted most strongly to inoculation with rhizobacteria; therefore, it can be considered the best gene candidate for assessing interactions between rhizobacteria and *M. truncatula*. Taking this into account, it seems likely that bacterial strains, in particular, *Pseudomonas brassicacearum* KK5 and *P. corrugata* KK7, can be used to induce the biosynthetic pathway of phenolic compounds in the model plant *M. truncatula* and thus in other legumes. This can result in the induction of resistance in plants and, consequently, improved disease control. The presented results allow us to focus future research on the impact of rhizobacteria on plant resistance by activating the phenylpropanoid biosynthesis pathway, and the role of individual phenolic compounds in disease control.

## Figures and Tables

**Table 1:** List of tested (white fields) and reference genes.

**Gene Name**	Gene Product Name	Locus Number	Sequence for PCR Primers (5′ → 3′)	Sequence for qPCR Primers (5′ → 3′)
*MtPAL 1*	phenylalanine ammonia-lyase 1	Medtr1g064090	TACCACTTCGCGGTACAATCAC TATGCTCCATAATAGCAGCAGCTT	not tested
*MtPAL 2*	phenylalanine ammonia-lyase 2	Medtr1g094780	GCTTCATTGGTTCTTTTTGATACAA TTCCTCCATGAAGTGCCTTATCC	CACTTCAGAAGCCCAAACAAGATAG TGTTGCGTATCGAATCACTTCAA
*MtPAL 3*	phenylalanine ammonia-lyase 3	Medtr2g094960	ACAAGATTAGCTTTGGCTGCT GCCACTTGACTCAGTCTTTTCT	GCGATGGCTGCATATTGTTCT TTATGCTGTTCAGCGCTTTGG
*MtPAL 4*	phenylalanine ammonia-lyase 4	Medtr5g098720	ACACCAATCTTGCCATTGCG TCCATAATAGCAGCTGCCTCG	AGCAGGTCTGAGTTCTGGGTTCT GAAGCCACACCAGATCCAACA
*MtPAL 6*	phenylalanine ammonia-lyase 6	Medtr7g101425	TACACGTTTGGCTCTCGCAT TGGCTACTTGACTTACGGTGT	not tested
*ACT*	actin 2	Medtr7g026230	TATCATAGATGGTTGGAACAGGACC GGAGACAGCCAGGACCAGC	TCAATGTGCCTGCCATGTATGT CTCACACCGTCACCAGAATCC

## Data Availability

The datasets generated during and/or analyzed during the current study are available from the corresponding author upon reasonable request.

## Supplementary Materials

The following supporting information can be downloaded at https://www.mdpi.com/article/10.3390/ijms252312684/s1.

## References

[1] Trivedi P., Leach J.E., Tringe S.G., Sa T., Singh B.K. (2020). Plant–Microbiome Interactions: From Community Assembly to Plant Health. Nat. Rev. Microbiol..

[2] Trivedi P., Batista B.D., Bazany K.E., Singh B.K. (2022). Plant–Microbiome Interactions under a Changing World: Responses, Consequences and Perspectives. New Phytol..

[3] Agler M.T., Ruhe J., Kroll S., Morhenn C., Kim S.-T., Weigel D., Kemen E.M. (2016). Microbial Hub Taxa Link Host and Abiotic Factors to Plant Microbiome Variation. PLoS Biol..

[4] Trivedi P., Mattupalli C., Eversole K., Leach J.E. (2021). Enabling Sustainable Agriculture through Understanding and Enhancement of Microbiomes. New Phytol..

[5] Naylor D., Coleman-Derr D. (2018). Drought Stress and Root-Associated Bacterial Communities. Front. Plant Sci..

[6] Berendsen R.L., Pieterse C.M.J., Bakker P.A.H.M. (2012). The Rhizosphere Microbiome and Plant Health. Trends Plant Sci..

[7] Whipps J.M. (2001). Microbial Interactions and Biocontrol in the Rhizosphere. J. Exp. Bot..

[8] Zhang S., Li C., Si J., Han Z., Chen D. (2022). Action Mechanisms of Effectors in Plant-Pathogen Interaction. Int. J. Mol. Sci..

[9] Kloepper J.W., Schroth M.N. (1981). Relationship of in Vitro Antibiosis of Plant Growth-Promoting Rhizobacteria to Plant Growth and the Displacement of Root Microflora. Phytopathology.

[10] Glick B.R., Holguin G., Patten C.L., Penrose D.M. (1999). Biochemical and Genetic Mechanisms Used by Plant Growth Promoting Bacteria.

[11] Bhattacharyya P.N., Jha D.K. (2012). Plant Growth-Promoting Rhizobacteria (PGPR): Emergence in Agriculture. World J. Microbiol. Biotechnol..

[12] Mendes R., Kruijt M., De Bruijn I., Dekkers E., Van Der Voort M., Schneider J.H.M., Piceno Y.M., DeSantis T.Z., Andersen G.L., Bakker P.A.H.M. (2011). Deciphering the Rhizosphere Microbiome for Disease-Suppressive Bacteria. Science.

[13] Martínez-Viveros O., Jorquera M.A., Crowley D.E., Gajardo G., Mora M.L. (2010). Mechanisms and Practical Considerations Involved in Plant Growth Promotion by Rhizobacteria. J. Soil Sci. Plant Nutr..

[14] Pieterse C.M.J., Zamioudis C., Berendsen R.L., Weller D.M., Van Wees S.C.M., Bakker P.A.H.M. (2014). Induced Systemic Resistance by Beneficial Microbes. Annu. Rev. Phytopathol..

[15] Shah A., Nazari M., Antar M., Msimbira L.A., Naamala J., Lyu D., Rabileh M., Zajonc J., Smith D.L. (2021). PGPR in Agriculture: A Sustainable Approach to Increasing Climate Change Resilience. Front. Sustain. Food Syst..

[16] Aroca R., Ruiz-Lozano J.M., Zamarreño Á.M., Paz J.A., García-Mina J.M., Pozo M.J., López-Ráez J.A. (2013). Arbuscular Mycorrhizal Symbiosis Influences Strigolactone Production under Salinity and Alleviates Salt Stress in Lettuce Plants. J. Plant Physiol..

[17] Choudhary D.K., Prakash A., Johri B.N. (2007). Induced Systemic Resistance (ISR) in Plants: Mechanism of Action. Indian J. Microbiol..

[18] Bakker P.A.H.M., Doornbos R.F., Zamioudis C., Berendsen R.L., Pieterse C.M.J. (2013). Induced Systemic Resistance and the Rhizosphere Microbiome. Plant Pathol. J..

[19] Pieterse C.M.J., Van der Does D., Zamioudis C., Leon-Reyes A., Van Wees S.C.M. (2012). Hormonal Modulation of Plant Immunity. Annu. Rev. Cell Dev. Biol..

[20] Van Loon L.C., Bakker P.A.H.M., Pieterse C.M.J. (1998). Systemic resistance induced by rhizosphere bacteria. Annu. Rev. Phytopathol..

[21] Yu Y., Gui Y., Li Z., Jiang C., Guo J., Niu D. (2022). Induced Systemic Resistance for Improving Plant Immunity by Beneficial Microbes. Plants.

[22] Daroodi Z., Taheri P., Tarighi S. (2021). Direct Antagonistic Activity and Tomato Resistance Induction of the Endophytic Fungus Acrophialophora Jodhpurensis against Rhizoctonia Solani. Biol. Control.

[23] Guo Q., Li Y., Lou Y., Shi M., Jiang Y., Zhou J., Sun Y., Xue Q., Lai H. (2019). Bacillus Amyloliquefaciens Ba13 Induces Plant Systemic Resistance and Improves Rhizosphere Microecology against Tomato Yellow Leaf Curl Virus Disease. Appl. Soil Ecol..

[24] Wang M., Xue J., Ma J., Feng X., Ying H., Xu H. (2020). Streptomyces Lydicus M01 Regulates Soil Microbial Community and Alleviates Foliar Disease Caused by Alternaria Alternata on Cucumbers. Front. Microbiol..

[25] Chakraborty U., Chakraborty B., Basnet M. (2006). Plant Growth Promotion and Induction of Resistance in Camellia Sinensis by Bacillus Megaterium. J. Basic Microbiol..

[26] Dixon R.A., Paiva N.L. (1995). Stress-Induced Phenylpropanoid Metabolism. Plant Cell.

[27] Vogt T. (2010). Phenylpropanoid Biosynthesis. Mol. Plant.

[28] Liu R., Xu S., Li J., Hu Y., Lin Z. (2006). Expression Profile of a PAL Gene from Astragalus Membranaceus Var. Mongholicus and Its Crucial Role in Flux into Flavonoid Biosynthesis. Plant Cell Rep..

[29] Singh K., Kumar S., Rani A., Gulati A., Ahuja P.S. (2009). Phenylalanine Ammonia-Lyase (PAL) and Cinnamate 4-Hydroxylase (C4H) and Catechins (Flavan-3-Ols) Accumulation in Tea. Funct. Integr. Genom..

[30] Hahlbrock K., Scheel D. (1989). Physiology and Molecular Biology of Phenylpropanoid Metabolism. Annu. Rev. Plant Biol..

[31] Gholami A., De Geyter N., Pollier J., Goormachtig S., Goossens A. (2014). Natural Product Biosynthesis in Medicago Species. Nat. Prod. Rep..

[32] Schwede T.F., Rétey J., Schulz G.E. (1999). Crystal Structure of Histidine Ammonia-Lyase Revealing a Novel Polypeptide Modification as the Catalytic Electrophile. Biochemistry.

[33] Purwar S., Sundaram S., Sinha S., Gupta A., Dobriyall N., Kumar A. (2013). Expression and in Silico Characterization of Phenylalanine Ammonium Lyase against Karnal Bunt (*Tilletia indica*) in Wheat (*Triticum aestivum*). Bioinformation.

[34] Rawal H.C., Singh N.K., Sharma T.R. (2013). Conservation, Divergence, and Genome-Wide Distribution of PAL and POX A Gene Families in Plants. Int. J. Genom..

[35] Wanner L.A., Li G., Ware D., Somssich I.E., Davis K.R. (1995). The Phenylalanine Ammonia-Lyase Gene Family in Arabidopsis Thaliana. Plant Mol. Biol..

[36] Raes J., Rohde A., Christensen J.H., Van de Peer Y., Boerjan W. (2003). Genome-Wide Characterization of the Lignification Toolbox in Arabidopsis. Plant Physiol..

[37] Tuskan G.A., Difazio S., Jansson S., Bohlmann J., Grigoriev I., Hellsten U., Putnam N., Ralph S., Rombauts S., Salamov A. (2006). The Genome of Black Cottonwood, Populus Trichocarpa (Torr. & Gray). Science.

[38] Bagal U.R., Leebens-Mack J.H., Lorenz W.W., Dean J.F.D. (2012). The Phenylalanine Ammonia Lyase (PAL) Gene Family Shows a Gymnosperm-Specific Lineage. BMC Genom..

[39] Dong C., Ning C.A.O., Zhang Z., Shang Q. (2016). Phenylalanine Ammonia-Lyase Gene Families in Cucurbit Species: Structure, Evolution, and Expression. J. Integr. Agric..

[40] Feng Y., Huang Q., Zhang R., Li J., Luo K., Chen Y. (2022). Molecular characterisation of PAL gene family reveals their role in abiotic stress response in lucerne (*Medicago sativa*). Crop Pasture Sci..

[41] Ren W., Wang Y., Xu A., Zhao Y. (2019). Genome-Wide Identification and Characterization of the Phenylalanine Ammonia-Lyase (PAL) Gene Family in Medicago Truncatula. Legume Res. Int. J..

[42] Handberg K., Stougaard J. (1992). Lotus Japonicus, an Autogamous, Diploid Legume Species for Classical and Molecular Genetics. Plant J..

[43] Tadege M., Wen J., He J., Tu H., Kwak Y., Eschstruth A., Cayrel A., Endre G., Zhao P.X., Chabaud M. (2008). Large-scale Insertional Mutagenesis Using the Tnt1 Retrotransposon in the Model Legume Medicago Truncatula. Plant J..

[44] Young N.D., Debellé F., Oldroyd G.E.D., Geurts R., Cannon S.B., Udvardi M.K., Benedito V.A., Mayer K.F.X., Gouzy J., Schoof H. (2011). The Medicago Genome Provides Insight into the Evolution of Rhizobial Symbioses. Nature.

[45] Roy B.A., Kirchner J.W. (2000). Evolutionary Dynamics of Pathogen Resistance and Tolerance. Evolution.

[46] Glazebrook J. (2005). Contrasting Mechanisms of Defense against Biotrophic and Necrotrophic Pathogens. Annu. Rev. Phytopathol..

[47] Huang J., Gu M., Lai Z., Fan B., Shi K., Zhou Y.-H., Yu J.-Q., Chen Z. (2010). Functional Analysis of the Arabidopsis PAL Gene Family in Plant Growth, Development, and Response to Environmental Stress. Plant Physiol..

[48] Kim D.S., Hwang B.K. (2014). An Important Role of the Pepper Phenylalanine Ammonia-Lyase Gene (PAL1) in Salicylic Acid-Dependent Signalling of the Defence Response to Microbial Pathogens. J. Exp. Bot..

[49] Monteón-Ojeda A., Mora-Aguilera J.A., Hernández-Castro E., Sandoval-Islas J.S., Azuara-Domínguez A., Damián-Nava A. (2022). Induction of Systemic Acquired Resistance Associated with the Enzyme Activity of Phenylalanine Ammonia-Lyase, Peroxidase, and Polyphenoloxidase and Its Effect on the Severity of Anthracnose on Nursery Mango Plants. Arch. Phytopathol. Plant Prot..

[50] Tonnessen B.W., Manosalva P., Lang J.M., Baraoidan M., Bordeos A., Mauleon R., Oard J., Hulbert S., Leung H., Leach J.E. (2015). Rice Phenylalanine Ammonia-Lyase Gene OsPAL4 Is Associated with Broad Spectrum Disease Resistance. Plant Mol. Biol..

[51] Liu Y., Liu L., Yang S., Zeng Q., He Z., Liu Y. (2019). Cloning, Characterization and Expression of the Phenylalanine Ammonia-Lyase Gene (PaPAL) from Spruce Picea Asperata. Forests.

[52] Wu Z., Gui S., Wang S., Ding Y. (2014). Molecular Evolution and Functional Characterisation of an Ancient Phenylalanine Ammonia-Lyase Gene (NnPAL1) from Nelumbo Nucifera: Novel Insight into the Evolution of the PAL Family in Angiosperms. BMC Evol. Biol..

[53] Shine M.B., Yang J., El-Habbak M., Nagyabhyru P., Fu D., Navarre D., Ghabrial S., Kachroo P., Kachroo A. (2016). Cooperative Functioning between Phenylalanine Ammonia Lyase and Isochorismate Synthase Activities Contributes to Salicylic Acid Biosynthesis in Soybean. New Phytol..

[54] Thomas J., Kim H.R., Rahmatallah Y., Wiggins G., Yang Q., Singh R., Glazko G., Mukherjee A. (2019). RNA-Seq Reveals Differentially Expressed Genes in Rice (*Oryza sativa*) Roots during Interactions with Plant-Growth Promoting Bacteria, Azospirillum Brasilense. PLoS ONE.

[55] Jain S., Vaishnav A., Varma A., Choudhary D.K. (2018). Comparative Expression Analysis of Defence-Related Genes in *Bacillus*-treated *Glycine max* upon challenge inoculation with selective fungal phytopathogens. Curr. Sci..

[56] Chen Y., Li F., Tian L., Huang M., Deng R., Li X., Chen W., Wu P., Li M., Jiang H. (2017). The Phenylalanine Ammonia Lyase Gene LjPAL1 Is Involved in Plant Defense Responses to Pathogens and Plays Diverse Roles in Lotus Japonicus-Rhizobium Symbioses. Mol. Plant-Microbe Interact..

[57] Chen T., Duan L., Zhou B., Yu H., Zhu H., Cao Y., Zhang Z. (2017). Interplay of Pathogen-Induced Defense Responses and Symbiotic Establishment in Medicago Truncatula. Front. Microbiol..

[58] Ali M.B., McNear D.H. (2014). Induced Transcriptional Profiling of Phenylpropanoid Pathway Genes Increased Flavonoid and Lignin Content in Arabidopsisleaves in Response to Microbial Products. BMC Plant Biol..

[59] Bhattacharyya C., Banerjee S., Acharya U., Mitra A., Mallick I., Haldar A., Haldar S., Ghosh A., Ghosh A. (2020). Evaluation of Plant Growth Promotion Properties and Induction of Antioxidative Defense Mechanism by Tea Rhizobacteria of Darjeeling, India. Sci. Rep..

[60] Singh R.P., Jha P.N. (2017). The PGPR Stenotrophomonas Maltophilia SBP-9 Augments Resistance against Biotic and Abiotic Stress in Wheat Plants. Front. Microbiol..

[61] Basha S.A., Sarma B.K., Singh D.P., Annapurna K., Singh U.P. (2006). Differential Methods of Inoculation of Plant Growth-Promoting Rhizobacteria Induce Synthesis of Phenylalanine Ammonia-Lyase and Phenolic Compounds Differentially in Chickpca. Folia Microbiol..

[62] Bahadur A., Singh U.P., Sarnia B.K., Singh D.P., Singh K.P., Singh A. (2007). Foliar Application of Plant Growth-Promoting Rhizobacteria Increases Antifungal Compounds in Pea (*Pisum sativum*) against Erysiphe Pisi. Mycobiology.

[63] Singh U.P., Sarma B.K., Singh D.P. (2003). Effect of Plant Growth-Promoting Rhizobacteria and Culture Filtrate of Sclerotium Rolfsii on Phenolic and Salicylic Acid Contents in Chickpea (*Cicer arietinum*). Curr. Microbiol..

[64] Jain A., Singh S., Kumar Sarma B., Bahadur Singh H. (2012). Microbial Consortium–Mediated Reprogramming of Defence Network in Pea to Enhance Tolerance against Sclerotinia Sclerotiorum. J. Appl. Microbiol..

[65] Singh A., Sarma B.K., Upadhyay R.S., Singh H.B. (2013). Compatible Rhizosphere Microbes Mediated Alleviation of Biotic Stress in Chickpea through Enhanced Antioxidant and Phenylpropanoid Activities. Microbiol. Res..

[66] Meena B., Radhajeyalakshmi R., Vidhyasekaran P., Velazhahan R. (2000). Effect of Foliar Application of Pseudomonas Fluoresencens on Activities of Phenylalanine Ammonia-Lyase, Chitinase and β-1,3–Glucanase and Accumulation of Phenolics in Rice. Acta Phytopathol. Entomol. Hung..

[67] Jasinski M., Banasiak J., Radom M., Kalitkiewicz A., Figlerowicz M. (2009). Full-Size ABC Transporters from the ABCG Subfamily in Medicago Truncatula. Mol. Plant-Microbe Interact..

[68] Edwards R., Kessmann H. (1992). Isoflavonoid Phytoalexins and Their Biosynthetic Enzymes. Mol. Plant Pathol..

[69] Bradford M.M. (1976). A Rapid and Sensitive Method for the Quantitation of Microgram Quantities of Protein Utilizing the Principle of Protein-Dye Binding. Anal. Biochem..

[70] Livak K.J., Schmittgen T.D. (2001). Analysis of Relative Gene Expression Data Using Real-Time Quantitative PCR and the 2−ΔΔCT Method. Methods.

