# Supplementary material for: Biosynthesis of Phenolic Compounds of Medicago truncatula After Inoculation with Selected PGPR Strains
Source: Int J Mol Sci. 2024 Nov 26;25(23):12684. doi: 10.3390/ijms252312684 (PMC11641612; doi:10.3390/ijms252312684)
Supplement: Supplementary file 1 [file ijms-25-12684-s001.zip › ijms-3300382-supplementary.pdf]

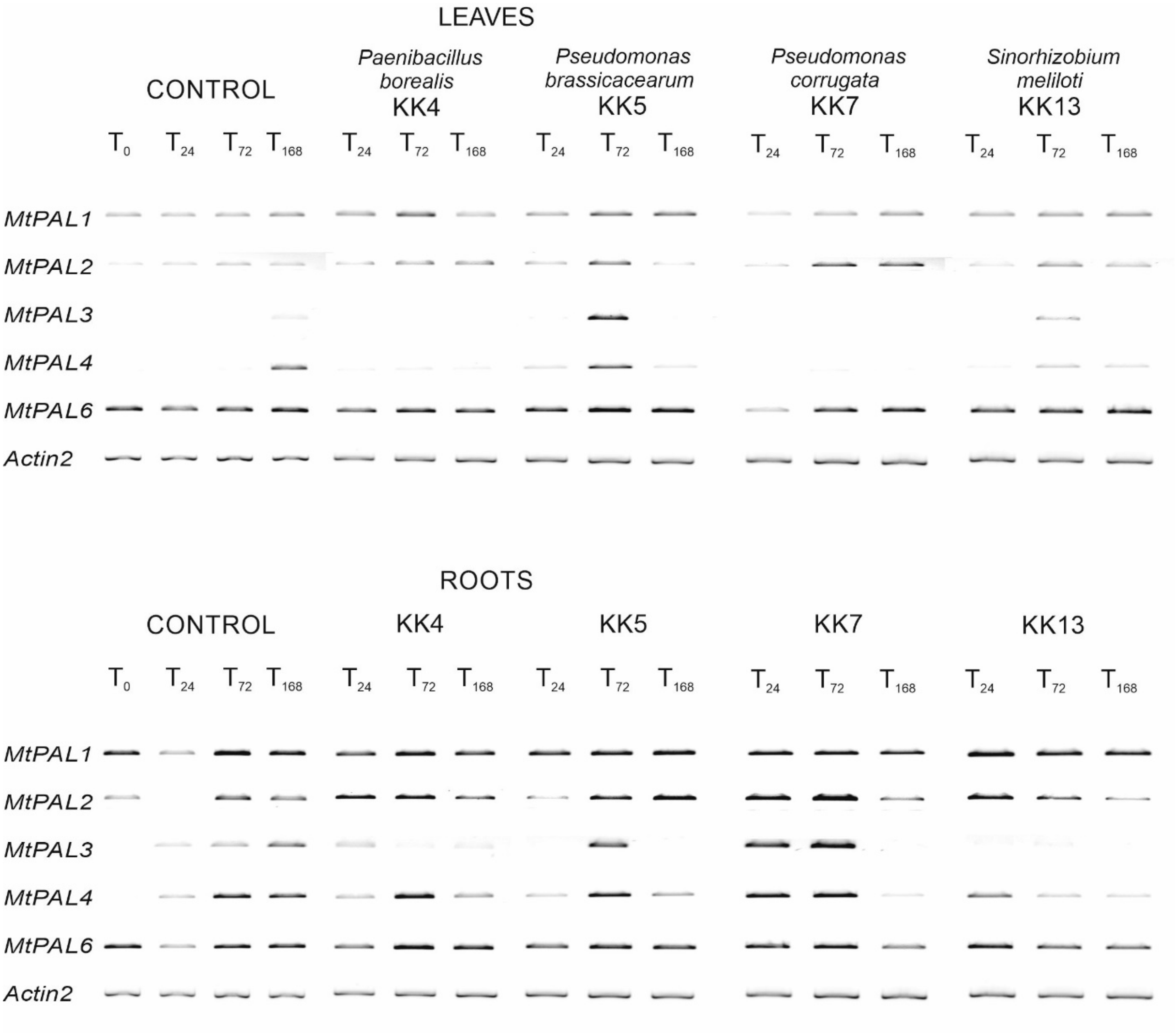

Figure S1 Semi-quantitative analysis of the expression of five genes encoding phenylalanine ammonia-lyase (*MtPAL*) in leaves and roots of *Medicago truncatula* seedlings just before (T0) and 24, 72 and 168h after inoculation with rhizobacteria.
